# Timekeeper's dilemma: How photo-thermal cues alter flowering duration

**DOI:** 10.1093/plphys/kiad240

**Published:** 2023-04-20

**Authors:** Kyle W Swentowsky, Janlo M Robil

**Affiliations:** Plant Physiology, American Society of Plant Biologists, USA; Cold Spring Harbor Laboratory, Cold Spring Harbor, NY 11724, USA; Plant Physiology, American Society of Plant Biologists, USA; Ateneo de Manila University, Loyola Heights, Quezon City 1108, Philippines

The future of agriculture will rely on humans’ ability to predict and modify nearly all aspects of plant growth and development to suit the diverse and changing conditions crops are cultivated in. Many plant traits, including inflorescence architecture, the number of lateral branches, and the floral transition, have been extensively studied, and their genetic control is well understood. However, other important traits such as flowering duration have received less attention, and their regulation is still mysterious. Following the floral transition when inflorescence meristems (IMs) are initiated, the plant experiences a period of time where it continues to produce IMs, and resources are allocated to the reproductive structures (González-Suárez et al. 2020). The reproductive phase will typically conclude in 1 of 2 ways: an annual plant will permanently senesce, and a perennial will transition back into a vegetative growth mode. The conclusion of the reproductive phase, therefore, represents an important decision for the plant. If the end of flowering is delayed, the plant will have more time to fill its reproductive structures with energy and nutrients, but this comes at the risk of missing the window for favorable environmental or ecological conditions (e.g. winter freeze or a seed disperser).

**Figure 1.**
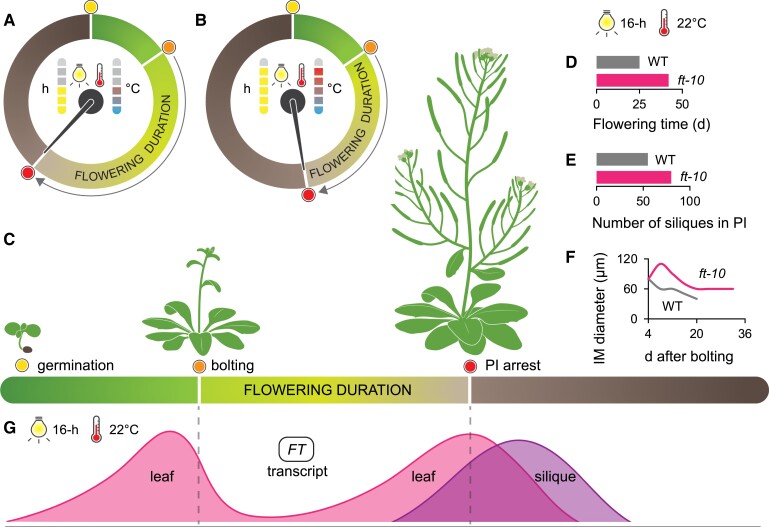
Photo-thermal cues regulate flowering duration in Arabidopsis. **A–B)**González-Suárez et al. (2023) found that increased photoperiods and temperatures shorten flowering duration as measured by the time between bolting and primary inflorescence (PI) arrest **(C)**. **D–F)** They also discovered that the *FLOWERING LOCUS T* (*FT*) plays a role in the response to photo-thermal cues because the *ft-10* mutant exhibits a longer flowering time, produces more siliques, and produces a larger inflorescence meristem (IM) under standard photo-thermal conditions. **G)** Furthermore, they discovered that *FT* expression in the leaf and silique peaks with PI arrest, adding to the previously known *FT* expression peak during the vegetative to floral transition. WT, wild type.

Despite its importance, we know little about the physiological and genetic control of flowering duration. In this issue of *Plant Physiology*, González-Suárez et al. (2023) provide the foundation for studying the genetics of inflorescence arrest in Arabidopsis. The authors first tested how the environment affects IM arrest and determined that long photoperiods (16 h of light) and high temperatures (20 to 27 °C) significantly shortened the flowering duration (Fig. 1A-C). Through a series of experiments with controlled temperature and photoperiod, the authors demonstrated that, regardless of what the plant experienced during the start of its life cycle, the flowering duration is affected by the temperature and photoperiod experienced only once flowering has been initiated. For these reasons, they conclude that IM arrest is under photo-thermal control during flowering. Careful observations revealed that the rate of floral primordia initiation is increased, and the IM diameter expands faster under higher temperatures and longer photoperiods. These findings indicate that the mechanism of the shortened flowering duration is caused by a faster progression of floral development.

Because photo-thermal cues are well known to affect the production of florigen, a signal that triggers plants to transition into reproductive growth, González-Suárez et al. (2023) tested whether photo-thermal floral arrest is under similar genetic control using a collection of Arabidopsis mutants. Mutants that were defective for genes involved in photoperiod-dependent flowering—*ft-1*, *ft-10*, *tsf-1*, *co-2*, and *gi-4*—also showed an increase in the reproductive duration. In contrast, *flm-3*, *flc-6*, and *svp-32*, mutants involved in temperature responsiveness, did not exhibit any differences in flowering duration. The largest effect on flowering duration was observed in mutants lacking the *FLOWERING LOCUS T* (*FT*) gene (Fig. 1D-F). These results indicate that although the flowering duration can be shortened in response to both elevated temperature and increased photoperiod, the photoperiodic regulator *FT* seems to be playing a major role in this process.

*FT* has been extensively characterized in Arabidopsis and is understood to encode the protein that acts as florigen (Corbesier et al. 2007), but it has conventionally not been considered to play a major role in plant development once flowering is initiated. To comprehend how this gene may affect the reproductive duration, leaf expression levels of the *FT* transcript were measured after flowering had initiated and were found to significantly increase over time and reach a peak at the time of flowering arrest (Fig. 1G). This increase in *FT* expression is enhanced in plants that were grown under warm conditions, thus establishing a solid correlation between *FT* expression and the decreased reproductive duration. In a key experiment, plants overexpressing FT showed a significantly reduced reproductive duration. Taken together, it is concluded that *FT* expression is enhanced under conditions that favor a shorter flowering duration, and *FT* is necessary and sufficient for this process.

The photo-thermal regulation of flowering duration described by González-Suárez et al. (2023) has implications for crop breeding and plant development as a whole. As future studies continue to characterize the genetic architecture that controls end of flowering, breeders could use this information to optimize the floral duration for productivity and enhanced growth in a particular environment. This study also adds to a growing body of literature supporting the idea that FT and its orthologous proteins have important roles in plant development outside of their conventional view as florigen. The authors propose a hypothesis through which FT is not simply a signal that instructs the plant to flower but an output of a “photo-thermal stopwatch” that regulates the timing of important developmental events in response to environmental cues.

Future work in this area may clarify this photo-thermal stopwatch hypothesis and focus on other aspects of photo-thermal IM arrest. For example, auxin and cytokinin are known to be involved in this process (Ware et al. 2020; Merelo et al. 2022; Walker et al. 2022), but their relationship with *FT* is unknown. This study could also provide context to how perennial plants are able to switch between vegetative and reproductive growth modes multiple times, whereas this transition is fixed in annual species.
